# Deregulated hyaluronan metabolism in the tumor microenvironment drives cancer inflammation and tumor-associated immune suppression

**DOI:** 10.3389/fimmu.2022.971278

**Published:** 2022-09-27

**Authors:** William Donelan, Paul R. Dominguez-Gutierrez, Sergei Kusmartsev

**Affiliations:** Department of Urology, University of Florida, College of Medicine, Gainesville, FL, United States

**Keywords:** tumor microenvironment, HYAL2, hyaluronan degradation, PD-L1, MDSC, tumor-associated macrophages, cancer inflammation, tumor-associated immune suppression

## Abstract

Hyaluronan (HA) is known to be a prominent component of the extracellular matrix in tumors, and many solid cancers are characterized by aberrant HA metabolism resulting in increased production in tumor tissue. HA has been implicated in regulating a variety of cellular functions in tumor cells and tumor-associated stromal cells, suggesting that altered HA metabolism can influence tumor growth and malignancy at multiple levels. Importantly, increased HA production in cancer is associated with enhanced HA degradation due to high levels of expression and activity of hyaluronidases (Hyal). Understanding the complex molecular and cellular mechanisms involved in abnormal HA metabolism and catabolism in solid cancers could have important implications for the design of future cancer therapeutic approaches. It appears that extensive crosstalk between immune cells and HA-enriched stroma contributes to tumor growth and progression in several ways. Specifically, the interaction of tumor-recruited Hyal2-expressing myeloid-derived suppressor cells (MDSCs) of bone marrow origin with HA-producing cancer-associated fibroblasts and epithelial tumor cells results in enhanced HA degradation and accumulation of small pro-inflammatory HA fragments, which further drives cancer-related inflammation. In addition, hyaluronan-enriched stroma supports the transition of tumor-recruited Hyal2^+^MDSCs to the PD-L1^+^ tumor-associated macrophages leading to the formation of an immunosuppressive and tolerogenic tumor microenvironment. In this review, we aim to discuss the contribution of tumor-associated HA to cancer inflammation, angiogenesis, and tumor-associated immune suppression. We also highlight the recent findings related to the enhanced HA degradation in the tumor microenvironment.

## Biology of hyaluronan

### Synthesis and degradation of HA

Hyaluronan, also called hyaluronic acid (HA), is a member of the glycosaminoglycan family of polysaccharides synthesized at the cell surface. Distributed widely through vertebrate connective, epithelial, and neural tissues, interactions between HA and the extracellular matrix (ECM) regulate cellular processes involved in embryonic development, tissue healing, inflammation, and tumor progression (1–3). HA is synthesized as an unbranched polymer of repeating disaccharides of glucuronic acid and *N*-acetylglucosamine. Normal physiological HA polymers consist of 2,000-25,000 disaccharides with molecular masses in the range of 10^6^ – 10^7^ kDa and polymer lengths of 2-25µm. HA is synthesized at the cell membrane as an unmodified polysaccharide by one of three hyaluronan synthases (HAS): HAS1, HAS2, or HAS3.

These transmembrane enzymes initiate synthesis on the inner side of the cell membrane and extrude HA onto the outer cell surface or into the ECM. HAS2 produces the largest HA polymers and is responsible for the majority of HA synthesis. Cell surface HA binding proteins, such as CD44 and RHAMM, anchor the matrix to the cell (4, 5). The degradation of HA is catalyzed by hyaluronidase enzymes (6, 7). Hyal1 and Hyal2 are the major hyaluronidases that are expressed in most tissues and hydrolyze the linkage between *N*-acetylglucosamine and glucuronic acid to generate HA fragments (8). Cell surface GPI-anchored Hyal2 degrades high molecular weight HA into intermediate molecular weight fragments of 20 kDa. In a concerted effort, HA fragments are further hydrolyzed by Hyal1 in endo-lysosomal compartments generating oligosaccharides.

### HA function and turnover in normal tissues and organs

HA has numerous biological functions both structurally and in terms of cell signaling. HA is an important constituent of the ECM that plays a role in the lubrication of joints and maintaining connective tissue integrity (1, 3). The biophysical properties of HA such as high hydration capacity contribute to tissue homeostasis and the structural integrity of the interstitial space which is critical for cellular remodeling. The transmembrane protein CD44 is considered the principal receptor for HA (2). CD44 expression is upregulated by growth factors and pro-inflammatory cytokines such as IL-1, epidermal growth factor (EGF), and transforming growth factor-beta (TGF-β). HA-CD44 interactions regulate key cellular functions including cell-cell adhesion, cellular migration, and receptor-mediated HA internalization and degradation. RHAMM is another key HA receptor that is present at the cell surface and also has isoforms that are localized to the cytoplasm and nucleus. HA-RHAMM interactions mediate cellular migration, cytoskeleton rearrangement, and intracellular signal transduction. HA-induced signal transduction *via* interactions with CD44 and RHAMM is mediated through a variety of cell signaling pathways including protein kinase C, focal adhesion kinase (FAK), mitogen-activated protein kinases (MAPKs), nuclear factor NF-κB, RAS, phosphatidylinositol kinase (PI3K), tyrosine kinases, and cytoskeletal components (1, 2). Under normal homeostatic conditions, the synthesis and degradation of HA are well balanced, but a shift towards increased degradation of HA occurs during pathological conditions (2).

## Hyaluronan metabolism in cancer

### Enhanced HA synthesis in tumors

Multiple studies have demonstrated that cancers are associated with elevated levels of HA, and human tumors typically have higher HA concentrations than in healthy tissues (2, 3). Human breast, lung, prostate, ovarian, nephroblastomas, and colon cancer are considered to be enriched with HA (1, 2). In these tumors, HA may support tumor growth by stimulating anchorage-independent growth and proliferation of tumor cells. Elevated HA levels have been identified in the urine of patients with bladder carcinomas (9). Not surprisingly, elevated expression of hyaluronan synthases have been identified in bladder cancer tissue and HAS1 is a predictor of tumor recurrence and disease-specific survival (10, 11). *In vivo* studies have confirmed that highly invasive and aggressive breast cancer cell lines express significantly elevated levels of HAS2 (12, 13). Additionally, clinical studies and experiments using mouse models have further found that expression of HAS2 in breast cancer tissues is associated with metastasis and reduced overall survival (14, 15). Enhanced HA synthesis and degradation are evident in the microenvironment of several tumor types and affect tumor growth, cell motility, and metastasis (2, 3).

In experimental tumor models, the overexpression of HA synthases leads to increased HA levels and accelerated tumor growth *in vivo*. The level of HA expression by tumors also correlates well with metastatic progression. For example, high levels of HA are found in the tumor stroma of patients with prostate cancer and are associated with metastasis (16, 17). Supporting these tumor-promoting functions of enhanced HA synthesis, experimental evidence demonstrates that suppression of HAS, reduction of HA synthesis using inhibitors, or use of HA binding molecules have been shown to impair tumor growth and reduce metastasis in several *in vitro* and *in vivo* models (3). Abnormal activation of CD44 and RHAMM cell signaling pathways can lead to malignant behavior, and HA interactions with cell surface receptors CD44 and RHAMM promote tumor progression by affecting cell proliferation, migration, and angiogenesis (2).

### Enhanced HA degradation in tumors

**Figure 1:**
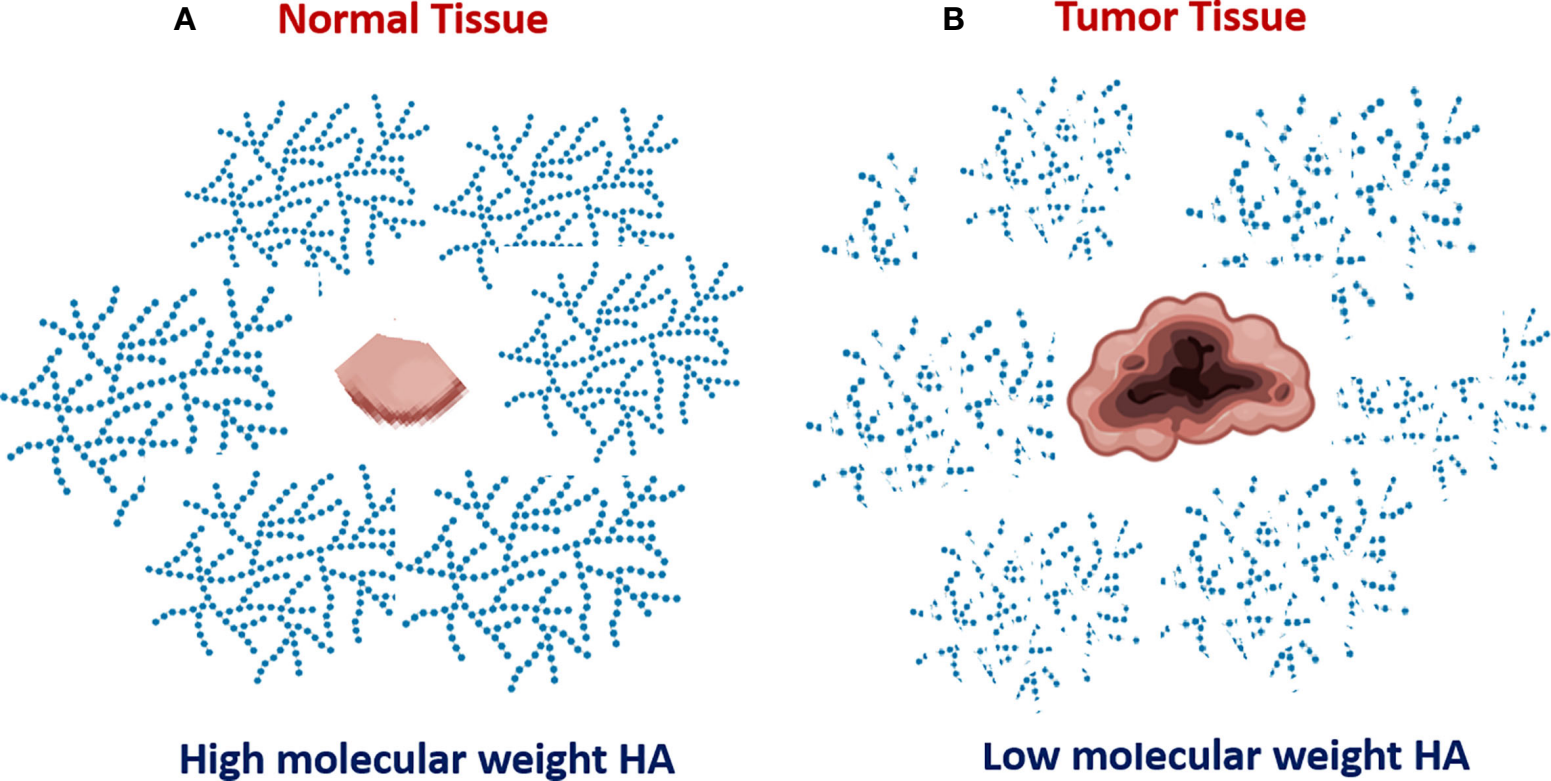
Increased degradation of extracellular hyaluronan in tumor tissue. Normal non-malignant bladder tissue produce HA with mostly high molecular weight **(A)**. In contrast, the tumor bladder tissue is characterized by enhanced HA degradation and accumulation of tumor-associated HA with low molecular weight **(B)**.

HA turnover is key to understanding the role of HA metabolism in tumor progression (1–3). HA is continually turned over in normal tissues, but the degradation products are rapidly cleared. In contrast, several types of cancer, including breast, prostate, bladder, melanoma, and lung cancers, are characterized by enhanced HA degradation (2, 3 and **Figure 1**). It is important to understand that HA degradation in the tumor microenvironment is a complex process that involves not only the tumor but also tumor-associated cells. The tumor stroma, which consists of an extracellular matrix and many non-cancer cell types contains an abundance of degraded HA compared to adjacent normal tissue. Bladder cancer progression is associated with enhanced expression of both Hyal2 and Hyal1 (11, 18), and Hyal2 gene expression is significantly increased in patients with progressive versus non-progressive bladder cancer. Elevated hyaluronidase expression and activity in tumor tissues lead to the accumulation of small HA fragments with low molecular weight.

### The dual nature of hyaluronan

The size of HA fragments is important for biological activity: high molecular-weight hyaluronan (HMW-HA), a major component of the extracellular matrix, is anti-oncogenic, which exerts anti-inflammatory and wound-healing activities. Evidence of anti-oncogenic properties of HMW-HA comes from the fact that naked mole rats show unusual resistance to cancer and the maximal lifespan of these mice exceeds 30 years (19). Analysis of HA produced by fibroblasts from naked mole rats revealed that its molecular size is five-time higher than human or mouse HA. Naked mole-rat tissues contain larger HA polymers and less detectable fragmentation than tissues of the more tumor-susceptible mouse. Knocking down the expression of hyaluronan synthase or overexpressing the HA-degrading enzyme, Hyal2, naked mole-rat cells become susceptible to malignant transformation and readily form tumors. A recent study demonstrated that at the molecular level, HMW-HA-mediated signaling through the CD44 receptor engages the tumor-suppressive Hippo pathway (20). Thus, recruitment of the polarity-regulating kinase PAR1b by the CD44 intracellular domain results in disruption of the Hippo signaling-inhibitory PAR1b-MST complex. Once liberated from PAR1b, MST activates Hippo signaling.

In contrast, the low-molecular-weight hyaluronan (LMW-HA) is pro-oncogenic. Thus, the HA fragments with low molecular weight produced in a hyaluronidase-dependent manner, inhibited Hippo signaling by competing with HMW-HA for CD44 binding (20). Tumor growth frequently is associated with enhanced HA degradation and the accumulation of small HA fragments with low molecular weight. The LMW-HA has been shown to promote tumor growth in a multifaceted manner by stimulating cancer inflammation, angiogenesis, and spreading of metastatic tumor cells (21–26).

## Effects of LMW-HA accumulation in cancer

### LMW-HA and cancer-related inflammation

Cancer-related inflammation is one of the hallmarks of tumor growth (27). Increased production of various inflammatory factors (chemokines, cytokines, bioactive lipids, etc) in tumor tissue supports constant mobilization and recruitment of immunosuppressive cells, precursors of endothelial cells, and cancer-associated fibroblasts which are needed for tumor growth. Increased degradation of hyaluronan in cancer tissue leads to the accumulation of HA fragments with small molecular weight, which is associated with elevated production of multiple cytokines, chemokines, and pro-angiogenic factors (1–3, 18, 28). Mechanistically, the LMW-HA elicits pro-inflammatory responses by modulating the toll-like receptor-4 (TLR-4) and activating the nuclear factor kappa B (NF-kB). In contrast, the HMW-HA manifests an anti-inflammatory effect by inhibiting NF-kB activation (29). During inflammation, HA can be degraded by hyaluronidases or cleaved by reactive oxygen species (ROS) (30–32). HA uptake and fragmentation by macrophages are thought to be important for the resolution of inflammation (24). The molecular weight of HA directly influences the immune macrophage activation (33, 34). According to these studies, the high-molecular-weight HA greater than 1,000 kDa exerts antiangiogenic, immunosuppressive, and anti-inflammatory effects that are important in wound healing, embryogenesis, and ovulation. In contrast, both medium-molecular weight HA and low-molecular-weight HA are pro-inflammatory, pro-angiogenic, and pro-tumor (34). HA added to LPS stimulated chondrocytes responded differently according to the HA molecular weight (32). Lee et al. demonstrated that HA fragments with molecular weight less than 50 kDa significantly increased iNOS production while medium MW-HA (1000 kDa) did not affect iNOS; however, HMW-HA (5000 kDa) significantly reduced iNOS in LPS-stimulated chondrocytes. A similar result was observed in LPS- stimulated macrophages where LMW-HA led to increases in iNOS, TNF-α, IL-6, IL-1β, TGF-β1, IL-10, IL-11, CCL2, and Arg1; however, in unstimulated macrophages, IL-10 significantly up-regulated by HMW-HA (24). Furthermore, LMW-HA increases several cytokines such as MMP-12, plasminogen activator inhibitor-1 (35, 36), CCL2 (MCP-1), CCL3 (MIP-1a), CCL4 (MIP-1β), keratinocyte chemoattractant, interleukins IL-8, and IL-12 by macrophages (37–39). Additionally, LMW-HA elicits the irreversible phenotypic and functional maturation of human dendritic cells (40, 41). In the model for acute lung injury to epithelium causes the production of inflammatory cytokines and chemokines resulting in the influx of neutrophils filled by macrophages to the site with further increase of cytokines and modulation of the extracellular matrix such as HA, collagen, fibronectin. Mechanistically, the low molecular weight HA fragments bind to the CD44, RHAMM, and Toll-like receptors  (5, 26, 42–44). Stimulation of TLR2/TLR4 results in enhanced production of inflammatory cytokines and chemokines. In addition, LMW-HA is a potent stimulant of arachidonic acid release in a time- and dose-dependent manner, inducing cPLA_2_α, ERK1/2, p38, and JNK phosphorylation, as well as activated COX2 expression and PGE_2_ production in primary human monocytes, murine RAW 264.7, and wild type bone marrow-derived macrophages. Specific cPLA_2_α inhibitors blocked HA-induced arachidonic acid release and PGE_2_ production in all of these cells (26). It is interesting to note that HMW-HA can significantly diminish TLR4, TLR2, MyD88, and NF-kB expression (45).

### LMW-HA and tumor angiogenesis

Multiple studies have demonstrated that both HMW-HA and LMW-HA are potent regulators of angiogenesis signaling, mainly by influencing endothelial cell (EC) behavior. One of the first studies demonstrated that partial degradation products of HA produced by the action of testicular hyaluronidase induced an angiogenic response, such as the formation of new blood vessels in the chick chorioallantoic membrane. Further fractionation of the digestion products showed that the activity was restricted to LMW-HA fragments between 4 and 25 disaccharides in length (46). More recently published studies indicate that LMW-HA stimulates vascular EC proliferation, migration, and tubule formation *in vitro*, as well as in various *in vivo* models of angiogenesis. In contrast, the HMW-HA displays anti-angiogenic properties by inhibiting EC proliferation, motility, and sprout formation (47–50). Since CD44 as well as RHAMM, two main receptors for HA, are present on the surface of the endothelial cells, both anti-RHAMM and anti-CD44 antibodies blocked the EC’s ability to form tubule-like structures in matrigel. Both CD44 and RHAMM stimulated by HA oligomers create a complex with ERK 1/2, which leads to the constitutive activation of ERK 1/2 and increased cell motility of invasive breast cancer cells (49). Similar ERK 1/2 pathway activation by LMW-HA has also been demonstrated in other tumor cell lines including ECs, such as human umbilical vein ECs (HUVECs), human microvessels endothelial cells, and human pulmonary artery ECs (49). Additionally, LMW-HA promotes the proliferation of HUVECs and ECs *via* ezrin, a linkage protein between the plasma membrane and actin skeleton that interacts with the cellular C-terminus of the CD44 receptor. In wound healing, CXCL12 stimulates angiogenesis by activating CXCR4 present on the EC surface and significantly increased cell migration, and induced faster-wound closure in wound closure assays. By pre-incubating with HMW-HA, the wound closure rate was significantly increased. *In vitro* studies have shown that CXCR4 activation by CXCL12 was significantly increased in HUVECs pretreated with HMW-HA; however, LMW-HA pre-incubation blocked CXCL12 signaling.

### LMW-HA and cancer metastasis

It appears that enhanced HA degradation is associated with tumor cell spreading. For example, accumulation of LMW-HA in tumor interstitial fluid correlates with lymphatic invasion and lymph node metastasis (51). Analysis of 176 serum specimens from breast cancer patients revealed (52) that level of serum LMW-HA but not total HA significantly correlated with lymph nodes metastasis, suggesting that serum LMW-HA may represent an indicator for metastasis development and prognosis for breast cancer progression. In addition, LMW-HA has been reported to induce cancer cell invasion by enhancing cancer cell motility (53).

Mechanistically, the HA receptors CD44s and RHAMM serve as mediators of HA-dependent development of metastasis (54). These receptors contribute to tumor progression *via* major pathways including the MAPK (MEK1, ERK1, 2) and SRC/FAK pathways that promote expression of an oncogenic transcriptome required for tumor cell survival, migration invasion, proliferation, and resistance to apoptosis (54).

CD44 co-localizes with HAS enzymes in lipid rafts where it is clustered by high molecular weight HA polymers and functions as a co-receptor for growth factor receptors to reduce the activation threshold of oncogenic driver signaling networks. For example, these small HA fragments limit oncogenic pathway activation and reverse drug resistance in CD133-positive highly tumorigenic subpopulations of ovarian carcinoma cells (55).

Another HA receptor, RHAMM is consistently overexpressed in many tumors, and its high expression is linked to the progression of multiple solid cancers. Increased expression of RHAMM is associated with castration-resistant disease in patients with prostatic metastases and elevated levels of both HA and RHAMM area are associated with a likelihood of biochemical failure in at-risk cancer patients after prostatectomy (56). Overexpression of RHAMM in breast primary cancer was linked to distant metastases (57), whereas increased RHAMM expression in colorectal cancers at the invasive front of primary tumors is linked to frequent lymphatic invasion, high tumor grade, and nodal metastasis (58).

## Mechanisms of HA degradation in the tumor microenvironment

The fragmented forms of HA occur in abundance in various malignancies. These small hyaluronan oligomers are assumed to be largely a result of increased hyaluronidase (Hyal) expression/activity (30). Functional perturbations of HA synthesis in cancer and degradation have revealed active roles of both the HA synthases and Hyals in epithelial tumor cells, stroma, tumor vascular formation, and resistance to chemotherapy (59). Six hyaluronidase-like gene sequences have been identified in humans: Hyal1-6 (6, 7), and two recently discovered enzymes with hyaluronidase activity, termed HYBID (KIAA1199; CEMIP) and TMEM (60, 61). Hyal1 and Hyal2 are the major hyaluronidases expressed in human somatic tissues. It has been proposed that Hyal1 and Hyal2 work together in a concerted effort (6, 62, 63). Hyal2 is a rate-limiting, glycosylphosphatidylinositol-linked (GPI-linked) enzyme that is anchored to the plasma membrane. It cleaves extracellular HMW-HA into intermediate size, 20-kDa HA fragments, or about 50 disaccharide units. The Hyal2-generated HA fragments are internalized, delivered to endosomes, and ultimately to lysosomes, where Hyal1 degrades the 20-kDa fragments to very small tetra-saccharides.

Importantly, both Hyal1 and Hyal2 contribute to intracellular and extracellular catabolism of hyaluronic acid, respectively, in a CD44-dependent manner (64). Several studies have shown that the HA can be internalized by normal, non-malignant cells (chondrocytes, macrophages, keratinocytes, etc.) for degradation and that the endocytosis is mediated *via* cell surface HA receptors. In cancer, the epithelial tumor cells frequently express the membrane-bound Hyal2 and can break down secreted HA into intermediate 20 kDa fragments. Analysis of tumor tissues from cancer patients and experimental animal tumors revealed that tumor cells frequently work together with tumor-recruited myeloid cells including myeloid-derived suppressor cells (MDSCs) and tumor-associated macrophages (TAMs) to break down the extracellular HA into small HA fragments (18, 65, 66). It appears, that tumor-associated myeloid cells have significant amounts of internalized HA and display higher levels of Hyal1 expression as compared to tumor cells. Accordingly, the tumor-associated myeloid cells, and particularly TAMs, are more efficient in breaking down the HMW-HA into small pro-inflammatory and pro-angiogenic HA fragments.

### Tumor stroma and hyaluronan metabolism

Tumor stroma supports tumor growth in multiple ways including simulation of proliferation, migration, invasion, promoting cancer-related inflammation, tumor angiogenesis, and also by contributing to tumor-associated immune suppression, resistance to cancer immunotherapy, and chemotherapy (67–72). Major cellular components of tumor stroma include cancer-associated fibroblasts (CAFs), TAMs, and mesenchymal and endothelial cells.

In addition to cells, the tumor stroma also has a highly complex extracellular component such as ECM, which comprises collagens, glycans, proteoglycans, and various secreted proteins such as growth and angiogenic factors, eicosanoids, chemokines, etc (73, 74). The state of tumor stroma is highly dynamic and is constantly being remodeled *via* the recruitment of myeloid cell subsets as well as precursors of endothelial and mesenchymal cells. HA is one of the major ECM components in tumor stroma. HA has been implicated in regulating a variety of cellular functions in both tumor cells and tumor-associated stromal cells, suggesting that altered HA levels can influence tumor growth and malignancy at multiple levels. Previously published studies have demonstrated that HA increases the proliferation rate of tumor cells *in vitro* and promotes cell survival under anchorage-independent conditions. Stromal HA accumulation is associated with a low immune response and poor prognosis in some cancers (75, 76) Due to the abnormal production of HA and its enhanced degradation in tumor tissues, it is plausible that aberrant HA metabolism in the tumor microenvironment is highly relevant for tumor growth and progression, specifically through stimulation of cancer inflammation, tumor angiogenesis, and modulation of the anti-tumor immune response. HA has frequently been implicated in monocyte/macrophage trafficking and activation. Pathology studies of human cancer specimens suggested that increased numbers of macrophages were correlated with HA accumulation in tumors (77). Also, it has been shown that TAMs preferentially infiltrate tumor tissues in an HA-dependent manner and concomitantly enhance neovascularization and tumor growth (78).

Recently we showed that HA-enriched tumor stroma directly contributes to the development of the immunosuppressive tumor microenvironment by supporting the formation of PD-L1-expressing macrophages (65). Thus, analysis of organotypic tumor tissue-slice cultures, from mice with implanted syngeneic tumors as well as from cancer patients, revealed that tumor-recruited myeloid cells directly interact with stromal cells to form the large PD-L1-expressing cell congregates. Using genetically modified tumor cells, we found that both epithelial tumor cells and vimentin-positive CAFs represent the major sources of HA in the tumor microenvironment. Furthermore, similar cell clusters comprised of HA-producing fibroblast-like cells and F4/80^+^PD-L1^+^ macrophages were detected in tumor-draining, but not in distant lymph nodes. Taking together, these findings indicate that the formation of multiple large HA-enriched stromal clusters that support the development of PD-L1-expressing antigen-presenting cells in the tumor microenvironment and draining lymph nodes could contribute to the immune escape and resistance to immunotherapy in cancer.

### Tumor-recruited Hyal2^+^ myeloid cells and degradation of tumor-associated hyaluronan

**Figure 2:**
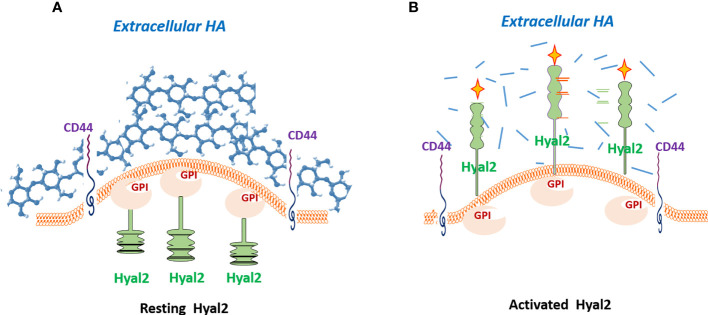
Hyal2 translocation is required for the degradation of extracellular hyaluronan. In a quiescent state, the Hyal2 molecule in myeloid cells resides predominantly in intracellular space **(A)**. However, activation of the CD44 signaling pathway results in translocation of Hyal2 to the cellular membrane, enabling the degradation of extracellular HA **(B)**. Depicted yellow crosses indicate the activated status of the enzyme Hyal2.

There are multiple pieces of evidence in various cancer types demonstrating that tumor-associated myeloid cells play pivotal roles in the formation of the immunosuppressive and tolerogenic tumor microenvironment that promotes immune escape and contributes to resistance to cancer immunotherapy. Tumors promote mobilization of myeloid-derived suppressor cells (MDSCs) using various mechanisms primarily from bone marrow and spleen (79, 80). Upon entering tumor tissue MDSCs directly interact with tumor cells. However, the mechanisms of these interactions are not fully understood. It appears that tumor-associated HA mediates the crosstalk between tumor-recruited Hyal2^+^ MDSCs, tumor cells, and stroma (64). Thus, Hyal2-expressing myeloid cells directly contact HA-producing tumor cells and CAFs. In a non-activated state, the HA-degrading enzyme Hyal2 in myeloid cells resides predominantly in intracellular space. However, upon activation in the tumor microenvironment Hyal2 translocate to the cell membrane, thus enabling the degradation of extracellular HA (**Figure 2**). The tumor-associated Hyal2-expressing myeloid cells have been detected in close contact with HA-producing CAFs and epithelial tumor cells, leading to enhanced degradation of tumor-associated fragmentation of HA in the tumor microenvironment. It should be noted that Hyal2 activity in myeloid cells is regulated by CD44 signaling and IL-1beta (18).

The increased numbers of Hyal2^+^ MDSCs have been detected in peripheral blood and tumor tissue in patients with bladder and kidney cancers (18, 66). In humans this co-express marker of myeloid cells CD11b and the marker of monocytic MDSCs CD33 (Hyl2^+^CD33^+^CD11b^+^). A similar subset of Hyal2-expressing MDSCs (Hyal2^+^Gr-1^+^CD11b^+^) has been found in tumor-bearing mice (65). Mobilization and tumor recruitment of Hyal2^+^ myeloid cells results in enhanced HA degradation in tumor tissue, leading to the accumulation of small HA fragments (~20 kDa) in the tumor microenvironment. In addition, the tumor-infiltrating myeloid cells show significantly higher expression of Hyal1 than tumor cells and have the ability to degrade HA into much smaller fragments with a molecular weight of less than 5 kDa (63). The smallest HA fragments exert the strongest pro-inflammatory and pro-angiogenic activities (2, 3, 7, 45).

**Figure 3:**
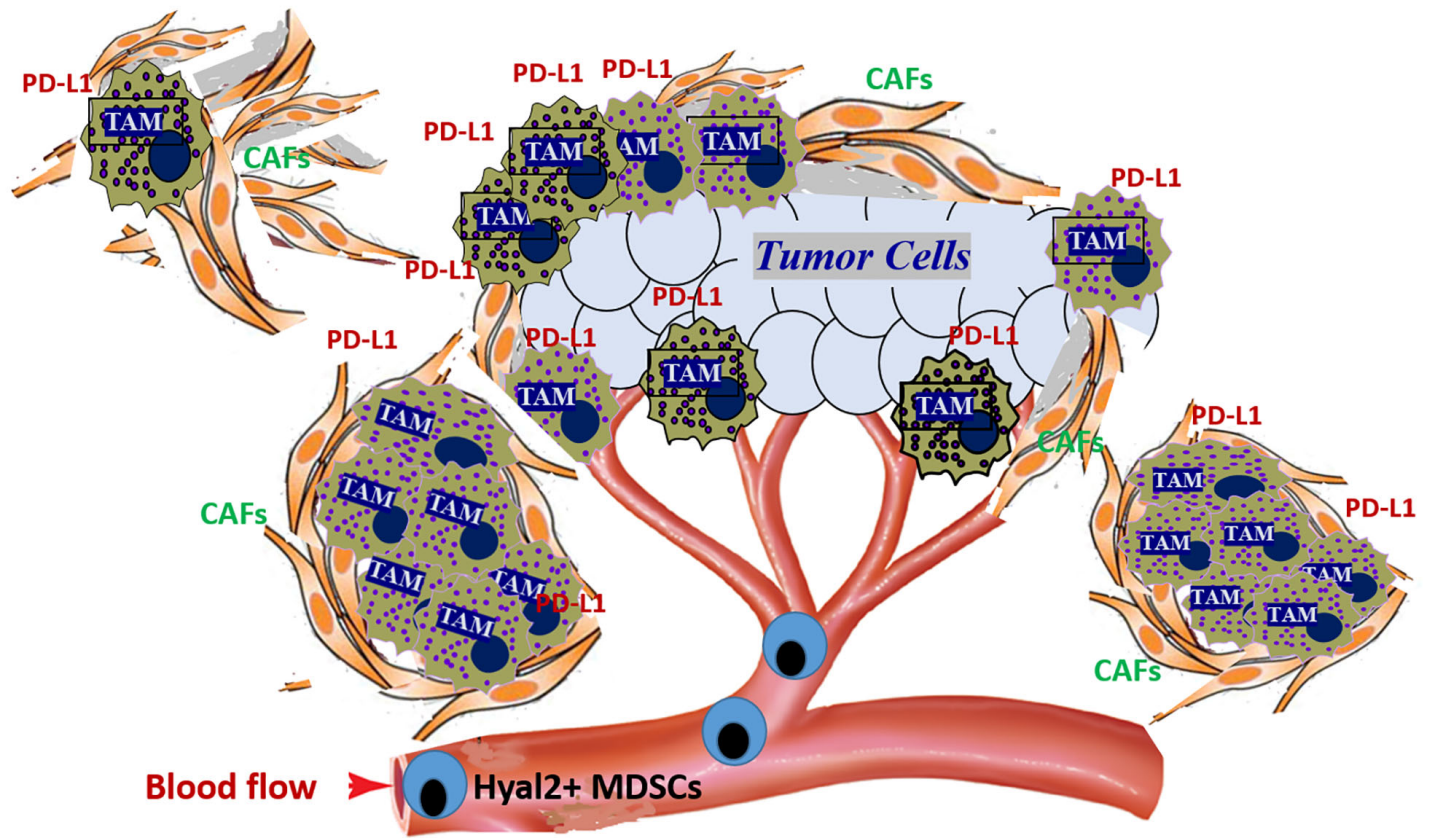
Tumor-recruited Hyal2^+^ MDSCs directly contribute to the development of the immunosuppressive tumor microenvironment by forming PD-L1^+^ macrophage clusters in the hyaluronan-enriched stroma. Tumors constantly secrete significant amounts of chemokines that attract the Hyal2^+^ MDSCs from bone marrow. Once recruited to the tumor, Hyal2-expressing myeloid cells start degradation of extracellular HA in the tumor microenvironment. Direct interaction of tumor-recruited myeloid cells with HA-producing cancer-associated fibroblasts (CAFs) and epithelial tumor cells leads to the accumulation of small HA pro-inflammatory and pro-angiogenic fragments. Hyal2^+^ MDSCs differentiate into immunosuppressive tumor-associated PD-L1^+^ macrophages (PD-L1^+^ TAMs), forming large PD-L1-expressing cell clusters in HA-enriched tumor stroma.

**Figure 4:**
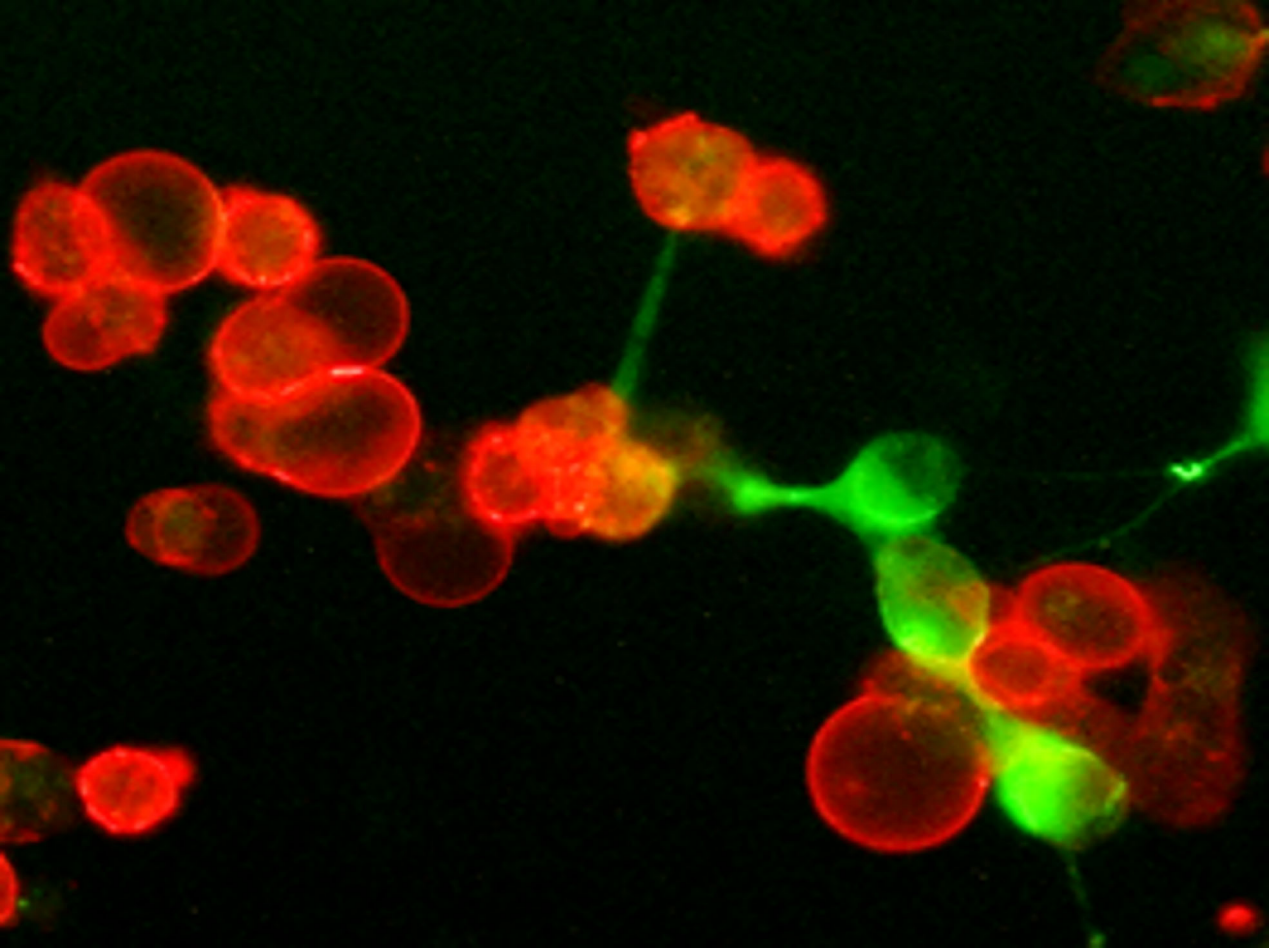
Tumor-associated hyaluronan directly supports the development of immunosuppressive PD-L1^+^ macrophages in the tumor microenvironment. Freshly prepared tumor tissue slices were cultured for twenty-four hours. Then cultures were fixed with 4% formaldehyde and stained PD-L1 (red) and HA (green). Representative IF image is shown.

Importantly, the tumor-recruited Hyal2^+^ MDSCs also co-express the immunosuppressive ligand PD-L1 (64, 65). In mouse tumor models, upon contact with HA-producing tumor cells or CAFs, Hyal2^+^ MDSCs can proliferate and differentiate into immunosuppressive macrophages, forming the large PD-L1^+^ myeloid cell clusters (**Figures 3** and **4**). Interestingly, during the transition of Hyal2^+^MDSCs into TAMs, the activity of HA-degrading activity of Hyal2 is reduced, while expression of immunosuppressive ligand PD-L1 is markedly up-regulated. It is still unclear how the degradation of extracellular HA promotes differentiation of Hyal2^+^Gr-1^+^PD-L1^+^ MDSCs into more mature immunosuppressive F4/80^+^PD-L1^+^ tumor-associated macrophages. It is plausible that under pro-inflammatory conditions in the tumor microenvironment small HA fragments promote the transition of MDSCs into activated immunosuppressive PD-L1^+^ macrophages. Collectively, these data indicate that tumor-recruited myeloid cells contribute to tumor growth through degradation of tumor-associated HA, promoting cancer-related inflammation, tumor angiogenesis, and tumor-associated immune suppression.

## Conclusions

Understanding the complex molecular and cellular mechanisms involved in abnormal HA metabolism and catabolism in solid cancers could have important implications for the design of future cancer therapeutic approaches. It appears that extensive crosstalk between tumor-associated myeloid cells and HA-enriched stroma contributes to the tumor growth and progression in several ways. Specifically, the interaction of tumor-recruited Hyal2-expressing myeloid cells with HA-producing stromal cells results in enhanced HA degradation and accumulation of small pro-inflammatory and pro-angiogenic HA fragments (18). HA-enriched stroma directly supports the development of PD-L1-expressing macrophages, thus contributing to the formation of the immunosuppressive, tolerogenic microenvironment by creating a PD-L1 shield and preventing T cell-mediated immune response *via* the PD1/PD-L1 pathway (65, 66). Constant mobilization of bone marrow-derived Hyal2^+^ MDSCs, CAFs, and endothelial cells contribute to the highly dynamic development of tumor stroma, enhances degradation of tumor-associated HA, and further promotes the tumor progression through stimulation of cancer-related inflammation, tumor angiogenesis, and tumor-associated immune suppression. Therefore, the normalization of HA metabolism in the tumor microenvironment could potentially provide a strong beneficial step for improving the efficacy of existing approaches to treat cancer, particularly for cancer immunotherapy.

## Data availability statement

The original contributions presented in the study are included in the article/supplementary materials. Further inquiries can be directed to the corresponding author.

## Author contributions

WD wrote review materials related to HA metabolism in normal and malignant tissues. PD-G wrote review sections related to the roles of LMW-HA in inflammation and angiogenesis. SK composed the article, wrote review materials related to mechanisms of HA degradation in tumor microenvironment and stroma, wrote abstract, conclusion and prepared Figures. All authors contributed to the article and approved the submitted version.

## Funding

This work was supported by James and Ester King Biomedical Research Program, Florida Health Department, award 8JKO5 and 1923 Fund to SK.

## Conflict of interest

SK is a founder of K-Lab Therapeutics.

The remaining authors declare that the research was conducted in the absence of any commercial or financial relationships that could be construed as a potential conflict of interest.

## Publisher’s note

All claims expressed in this article are solely those of the authors and do not necessarily represent those of their affiliated organizations, or those of the publisher, the editors and the reviewers. Any product that may be evaluated in this article, or claim that may be made by its manufacturer, is not guaranteed or endorsed by the publisher.
